# The Chromatin Remodeler HELLS: A New Regulator in DNA Repair, Genome Maintenance, and Cancer

**DOI:** 10.3390/ijms23169313

**Published:** 2022-08-18

**Authors:** Estanislao Peixoto, Asad Khan, Zachary A. Lewis, Rafael Contreras-Galindo, Wioletta Czaja

**Affiliations:** 1 The Hormel Institute, University of Minnesota, Austin, MN 55912, USA; 2 Department of Microbiology, University of Georgia, Athens, GA 30602, USA; 3 Department of Plant Biology, University of Georgia, Athens, GA 30602, USA; 4 Department of Genetics, University of Georgia, Athens, GA 30602, USA; 5 Department of Genetics, University of Alabama, Birmingham, AL 35294, USA

**Keywords:** DNA damage, DNA repair, ATP-dependent chromatin remodelers, heterochromatin, HELLS, LSH, cancer

## Abstract

Robust, tightly regulated DNA repair is critical to maintaining genome stability and preventing cancer. Eukaryotic DNA is packaged into chromatin, which has a profound, yet incompletely understood, regulatory influence on DNA repair and genome stability. The chromatin remodeler HELLS (helicase, lymphoid specific) has emerged as an important epigenetic regulator of DNA repair, genome stability, and multiple cancer-associated pathways. HELLS belongs to a subfamily of the conserved SNF2 ATP-dependent chromatin-remodeling complexes, which use energy from ATP hydrolysis to alter nucleosome structure and packaging of chromatin during the processes of DNA replication, transcription, and repair. The mouse homologue, LSH (lymphoid-specific helicase), plays an important role in the maintenance of heterochromatin and genome-wide DNA methylation, and is crucial in embryonic development, gametogenesis, and maturation of the immune system. Human HELLS is abundantly expressed in highly proliferating cells of the lymphoid tissue, skin, germ cells, and embryonic stem cells. Mutations in HELLS cause the human immunodeficiency syndrome ICF (Immunodeficiency, Centromeric instability, Facial anomalies). HELLS has been implicated in many types of cancer, including retinoblastoma, colorectal cancer, hepatocellular carcinoma, and glioblastoma. Here, we review and summarize accumulating evidence highlighting important roles for HELLS in DNA repair, genome maintenance, and key pathways relevant to cancer development, progression, and treatment.

## 1. Regulation of DNA Repair in the Context of Chromatin

Maintenance of genome stability is critical for proper cell function, viability, tissue homeostasis, and cancer suppression. Indeed, significant levels of genomic instability are frequently associated with cancer [1,2]. The DNA molecules in nearly every cell of the body are constantly damaged by a variety of genotoxic agents originating from external (e.g., UV radiation, environmental carcinogens, chemotherapy drugs) and intracellular (by-products of cellular metabolism, inflammation) sources. Cells safeguard genomic stability by employing robust and tightly regulated DNA repair mechanisms involving base excision repair (BER), nucleotide excision repair (NER), homologous recombination (HR), and nonhomologous end joining (NHEJ). DNA repair dysregulation is one of the key drivers contributing to genome instability, malignant transformation, cancer progression, and metastasis and plays a central role in therapeutic responses [3].

In eukaryotes, the genome is packaged with histone proteins into nucleosomes and higher-order structures, creating structurally and functionally distinct chromatin domains. Chromatin packaging and dynamic reorganization via nucleosome remodeling have a profound, yet incompletely understood regulatory influence on DNA repair, replication, and genome maintenance. Indeed, packaging of DNA into nucleosomes and assembly of higher order chromatin structures restrict access of repair factors to damaged DNA templates and substantially inhibits DNA repair processes [4,5,6]. Therefore, proficient responses to genotoxic stress in the chromatin require dynamic chromatin reorganization to facilitate coordinated actions of key DNA repair pathways to safeguard genome stability in support of the proper cellular function and tissue homeostasis [7,8].

Over the past decade, significant effort has been made towards understanding how genome stability is maintained in the context of nuclear chromatin. Several studies in yeast and human cells revealed that SNF2 ATP-dependent chromatin remodelers (ACR) play important roles in genome maintenance by facilitating and integrating various steps in the DNA damage response, including DNA accessibility, damage signaling, and repair [4,9]. Conserved from yeast to human, canonical ACR enzymes, such as SWI/SNF, INO80, and ISWI, use ATP energy to alter chromatin structure and facilitate DNA accessibility by nucleosome sliding, ejection, and incorporation of histone variants; they play important roles in modulation of DNA repair, replication, and transcription. The human SWI/SNF remodeler is well established tumor suppressor. SWI/SNF is mutated in ~25% of all human cancers and plays an active role in driving oncogenic programs. Exploiting vulnerabilities of SWI/SNF has emerged as a promising therapeutic strategy in many cancer types [10,11]. Therefore, understanding the ACR-mediated regulation of DNA repair holds promise for the identification of new preventive and therapeutic strategies in cancer.

## 2. HELLS Belongs to the SNF2 Family of ATP-Dependent Chromatin Remodelers

HELLS (helicase, lymphoid specific; also known as SMARCA6, LSH, PASG) is a member of the SNF2 family of ACR. Mammalian HELLS has orthologues in plants (DDM1) and lower eukaryotes, including the yeast *Saccharomyces cerevisiae* (IRC5) and *Neurospora crassa* (MUS-30). HELLS is abundantly expressed in highly proliferating cells of the lymphoid tissue (thymus, bone marrow), skin, germ cells, and embryonic stem cells [12]. Cells deficient in HELLS are characterized by increased levels of genome instability, senescence, and sensitivity to genotoxic agents, phenotypes frequently associated with deficient DNA repair pathways. Mutations in HELLS cause human ICF syndrome (Immunodeficiency, Centromeric instability, Facial abnormalities), a rare autosomal recessive disorder characterized by severe immunodeficiency, developmental abnormalities, and mental retardation [13]. HELLS is frequently mutated or overexpressed in many types of cancer. The murine orthologue of HELLS is LSH (lymphoid-specific helicase). The complete loss of mouse LSH protein (LSH^−/−^ null) leads to postnatal lethality [14]. Partial deletion resulting in a low level of truncated LSH protein is sufficient to support short-term survival, but with severe growth retardation and a premature aging phenotype associated with global DNA hypomethylation and replicative senescence [15]. LSH associates with heterochromatin and is required for maintenance of the global DNA methylation patterns and heterochromatin structure [16,17,18]. LSH has also been implicated in preserving cellular identity, independent of DNA methylation, through genome-wide chromatin remodeling achieved by modulating nucleosome positioning [18]. Studies in fungi (*Saccharomyces* and *Neurospora*) and plants suggest that orthologues of HELLS are involved in protecting cells from DNA damage–induced cell death and genome instability [19,20,21,22].

Although mammalian HELLS remodelers have been implicated in DNA repair and genome maintenance, the molecular function of these proteins in modulating specific steps and DNA repair pathways is not well understood. Unlike most of the SNF2 ACRs, HELLS ATPase does not appear to associate with other proteins into stable multisubunit complexes. However, recent studies have reported that the CDCA7 protein is essential for loading HELLS onto chromatin, forms a bipartite complex with HELLS, and substantially stimulates the ATP-dependent nucleosome remodeling activity of HELLS in vitro [23].

## 3. HELLS Is a Key Epigenetic Regulator of Heterochromatin

Heterochromatin constitutes about one-third of the human genome, and its dysfunction is linked to genome stability and many aspects of tumorigenesis [24]. Prominent pericentromeric heterochromatin domains extend for several megabases on each chromosome and are composed of tandemly arrayed satellite repeats as well as dispersed transposons and retrovirus sequences [25]. The DNA of the centromeric regions is heavily methylated, which is necessary to maintain the compacted and silenced state of these domains. Pericentromeric heterochromatin is critical for proper kinetochore assembly, faithful segregation of chromosomes during cell division, suppression of mobile genetic elements, and preservation of the global genomic integrity. Defects in the structure of centromeric heterochromatin can result in aneuploidy and chromosomal abnormalities, which are found in many cancer types. The HELLS remodeler emerged as an important regulator of the centromeric chromatin [26]. Cells derived from ICF4 patients (carrying a mutation in the *HELLS* gene) display characteristic hypomethylation in pericentromeric repeats and centromeric satellite repeats, but not in subtelomeric regions [27]. Murine LSH was shown to be crucial for controlling pericentromeric heterochromatin stability by maintaining CpG methylation at these sites [17,28]. In addition, HEK293 cells carrying ICF HELLS mutations displayed substantial hypomethylation of centromeric alpha satellites and pericentromeric repeats, and significant accumulation of γH2AX signals co-localizing with the centromeric regions [29].

The repetitive nature of the pericentromeric DNA sequence and condensed pericentromeric heterochromatin pose significant challenges to DNA damage repair and DNA replication. Indeed, human centromeres are highly susceptible to breakage under the conditions of DNA damage–induced replication stress [30]. Deficient or aberrant repair within centromeric repeats can trigger improper recombination between the repetitive sequences, leading to the loss or gain of repeats that may significantly impact centromere structure, chromosome segregation, and genome stability [30]. It has been proposed that deregulation of DNA repair within heterochromatin is likely one of the major drivers of global genome instability and tumorigenesis [24]. Accumulating evidence suggests that HELLS modulates DNA repair within heterochromatin [31]. Recent studies describe direct involvement of HELLS in the repair of DNA double-strand breaks (DSBs) within the heterochromatin of G2/M phase of cell cycle [31,32]. Additionally, heterochromatin hypomethylation resulting from the loss of HELLS can lead to reactivation of transcription of the pericentromeric satellite repeats and formation of aberrant DNA:RNA hybrids (R-loops). Studies by Unoki et al. revealed that the HELLS/CDCA7 complex protects genome stability by regulating maintenance of DNA methylation, the resolution or prevention of DNA:RNA hybrids (R-loops), and DNA repair at pericentromeric satellite repeats. In addition, mouse LSH was found to be an important regulator of meiotic kinetochore function, histone H3/Thr3 phosphorylation, and centromere transcription during oocyte meiosis. LSH was enriched at meiotic kinetochores, and its targeted deletion induced centromere instability and abnormal chromosome segregation in mouse oocytes [33].

## 4. Genome Instability Associated with HELLS Deficiency

Loss of the HELLS is associated with various forms of genomic instability (Table 1). Depletion of HELLS/LSH in human fibroblasts and lipopolysaccharide-stimulated mouse lymphocytes results in elevated numbers of spontaneous ɣH2AX foci indicative of increased endogenous DNA damage [31]. HEK293 cells carrying ICF HELLS mutations showed increased apoptosis, abnormal chromosome segregation, aneuploidy, and significant accumulation of ɣH2AX signals in centromeric and telomeric regions [29].

Depletion of HELLS in human cells is also associated with an increase in spontaneously occurring micronuclei, a prominent marker of genomic instability [31]. Increased sister chromatid exchange (SCE) is often associated with chromosome instability. SCE is mediated by HR and involves breaking, exchange, and rejoining of the DNA segments between two sister chromatids during DNA replication. It has been demonstrated that replication stress can generate unrepaired breaks and can lead to increased levels of SCE at fragile sites [39]. Elevated SCE was reported in lipopolysaccharide-stimulated mouse lymphocytes depleted of LSH, suggesting that loss of LSH leads to elevated replication stress and SCE [36]. By contrast, another study reported that HELLS-depleted HeLa cells showed fewer ionizing radiation (IR)-induced SCEs, but no change in spontaneous SCE, as compared with the control cells, a phenotype that mimics downregulation of ATM or Artemis, which also produce lower IR-induced SCE but no change in spontaneous SCE [31]. The discrepancies in levels of SCE might be attributed to different cell types, mouse lymphocytes vs human cancer cells, and involvement of HELLS in different aspects of DNA repair and genome maintenance.

## 5. HELLS Modulates Multiple DNA Repair Pathways

The chromatin-based response to replication stress or DSBs involves rapid phosphorylation of serine 139 on histone H2AX. The phosphorylated form, known as ɣH2AX, is a well-established marker of DSBs and stalled replication forks [40,41]. A study by Burrage et al. [32] showed that the HELLS remodeler was important for promoting phosphorylation of ɣH2AX and efficient repair of DSBs in human and mouse fibroblasts in response to IR-induced DNA damage [32]. LSH-deficient mouse embryonic fibroblasts (MEFs) displayed ~50% less ɣH2AX in response to IR, as measured by immunofluorescence and western blotting. LSH-deficient mouse and human fibroblasts had reduced proliferation rates and viability after exposure to IR, and lower capacity (by ~50%) to repair DSBs as measured with a comet assay. Further, deficient phosphorylation of ɣH2AX was associated with reduced recruitment of MDC1 and 53BP1 to DSB and compromised phosphorylation of the checkpoint kinase CHK2, all key effectors of DNA damage signaling. Importantly, it was also reported that reduction of ɣH2AX in HELLS-deficient cells was independent of DNA methylation levels in mouse and human fibroblasts, given that the MEFs null for the maintenance DNA methyltransferase DNMT1 displayed normal ɣH2AX kinetics in response to gamma radiation [32].

Another study, by Kollárovič et al. [31], showed that HELLS facilitates HR and DSB repair specifically in heterochromatin of G2/M cells, and promotes genome stability in both undamaged cells and cells exposed to IR. The siRNA-mediated depletion of HELLS in human fibroblasts was associated with increased spontaneous ɣH2AX foci and micronuclei formation. HR is initiated with break recognition and initiation of end-resection by the MRE11-RAD50-NBS1 complex in association with CtIP. HELLS promoted repair of DSBs within G2 heterochromatin by interacting with CtIP and promoting its accumulation at IR-induced breaks. This study concluded that HELLS is not an essential core HR factor for repair of all DSBs, but rather was specific to a subset of DSBs localized within the heterochromatin of G2/M cells [31].

Mammalian NHEJ is the primary pathway for the repair of DSBs throughout the cell cycle. NHEJ plays an important role in the maturation of the immune system by promoting physiological DSBs repair during V(D)J recombination in the early stages of B and T cell maturation [42]. Defects in NHEJ cause immunodeficiency and are associated with growth retardation and neurodevelopmental abnormalities [43]. HEK293 cells carrying ICF HELLS mutations had compromised canonical (C)-NHEJ and a delay in XRCC5 accumulation at DNA damage sites. The defect in C-NHEJ in HELLS-deficient cells was also associated with increased apoptosis, abnormal chromosome segregation, aneuploidy, and significant accumulation of ɣH2AX signals in centromeric and telomeric regions [29].

Proficient NHEJ is necessary for the adaptive immune response involving generation of diverse antigen receptors in T cells, as well as for B-cell class switch recombination (CSR), which contributes diverse isotypes of immunoglobulins to respond to and fight diverse pathogens [44]. HELLS-deficient purified B cells produce fewer immunoglobulins, and high-throughput sequencing analyses of CSR junctions and a chromosomal break repair assay revealed impairments in the NHEJ pathway in HELLS-deficient B cells [38].

Mammalian HELLS/LSH has orthologues in lower eukaryotes and plants, including the yeast *Saccharomyces cerevisiae* (IRC5), the filamentous fungus *Neurospora crassa* (MUS-30), and *Arabidopsis thaliana* (DDM1). IRC5, MUS-30, and DDM1 have been implicated in the cellular responses to alkylation-induced DNA damage and genome maintenance [19,20,21]. Studies in yeast showed that IRC5 is required for completion of alkylated DNA replication [20,21]. Studies in *N. crassa* suggest that MUS-30 might be important for stabilization of replication forks stalled by damaged DNA or repair intermediates [19], and MUS-30 is essential for preserving genome stability and supporting cell survival in response to alkylation DNA damage [19]. However, MUS-30 is not required for DNA methylation in *Neurospora* or for DSB repair by HR or NHEJ [19]. Whether MUS-30 regulates DSB repair within a subset of specific genomic loci or within heterochromatin domains remains to be determined [19]. Plant cells deficient in DDM1 demonstrated increased sensitivity to the DNA alkylating agent methyl methanesulfonate (MMS) and impaired BER and NER capacity as measured by in vitro repair assays with cell extracts [22].

## 6. HELLS in DNA Replication

Faithful DNA replication is critical to maintaining genome integrity. DNA replication is carried out by replication forks (replisomes) fired from the origins of replication. Replisomes contain a multitude of additional associated proteins that are actively involved in chromatin disassembly in front of the fork, DNA unwinding, DNA synthesis, and chromatin reassembly after the fork. Additional factors facilitate progression of the replication forks through difficult-to-replicate regions of the genome, such as compacted and repeat-rich heterochromatin. Defects in the progression of the replication fork can lead to fork stalling, collapse, and generation of DSBs, which if not repaired properly can lead to gross chromosomal breaks and rearrangements or cell death. A recent study by Xu et al. [36] found a new role for HELLS in replication fork stability and restart in response to hydroxyurea-mediated replication stress [36]. MRE11 and EXO1 can degrade nascent DNA at a stalled fork, leading to DNA breaks at the fork [36]. The protection of stalled forks is mediated by the macroH2A histone variant. HELLS prevents nucleolytic degradation of the stalled fork by promoting deposition of macroH2A at the replication fork. The proposed model suggests that, in HELLS-proficient cells, nucleosomes with macroH2A are abundant at the replication forks, which creates a chromatin environment supportive of BRCA1/BARD1 recruitment. BRCA1 promotes RAD51 filament formation, displaces RPA2, and protects nascent DNA from nucleolytic degradation by MRE and EXO1. Depletion of HELLS leads to reduced macroH2A occupancy and alters the chromatin environment at stalled replication forks. This enables recruitment of 53BP1, which prevents BRCA1 recruitment and results in persistent RPA2, lack of RAD51 filament formation, and increased nucleolytic degradation of the stalled forks.

## 7. HELLS Dysregulation in Cancer

HELLS has been implicated in several cancer pathways, associated with proliferative signaling, genome instability, deregulated cell energetics, and invasion. Genome instability (mutations, chromosomal rearrangements, aneuploidy) is a prominent hallmark of nearly all types of cancer (~90%). Importantly, genome instability is strongly associated with cancer heterogeneity, progression, metastasis, and poor survival [1,2]. Deficient and/or dysregulated DNA repair or DNA replication are the key pathways contributing to genome instability. Chromatin remodeling is a critical component of efficient DNA repair, genome stability and therapeutic responses.

Mutations or overexpression of HELLS have been associated with many types of cancer, including leukemia, retinoblastoma, colorectal cancer, hepatocellular carcinoma (HCC), and glioblastoma [37,45,46,47]. Contribution of HELLS to tumorigenesis appears to be frequently mediated through epigenetic and transcriptional mechanisms, and it remains to be determined whether HELLS influences aspects of carcinogenesis and therapeutic responses by regulating DNA repair pathways and genomic stability.

HELLS has been implicated in leukemogenesis because of the high incidence of an alternative mRNA transcript containing a 75-nt deletion in samples of acute myeloid leukemia and acute lymphocytic leukemia [12]. Recent studies report a role for HELLS in orchestrating a transcriptional program sustaining neoplastic features in anaplastic lymphoma kinase negative anaplastic large cell lymphoma [48].

HELLS has been reported to be a key epigenetic driver of HCC. Overexpression of HELLS in HCC is associated with increased nucleosome occupancy, which mediates the silencing of multiple tumor suppressor genes, including E-cadherin, *FBP1*, *IGFBP3*, *XAF1*, and *CREB3L3*, thereby promoting HCC progression [34]. Inactivation of HELLS in HCC cells reduces tumor growth, decreases metastasis and metabolic reprogramming, and reverses the Warburg effect (alternative metabolism leading to the production of lactate instead of CO_2_) [34].

Retinoblastoma is an aggressive childhood cancer initiated by the bi-allelic inactivation of *RB1* in developing retina. A recent study reported HELLS to be critical for retinoblastoma tumor initiation and progression [47]. Transcriptional expression of HELLS is regulated by the retinoblastoma RB/E2F pathway. Inactivation and loss of RB1 results in overexpression of HELLS and increased proliferation of retinoblastoma tumors; HELLS has therefore emerged as a potential new therapeutic target for retinoblastoma [47,49]. In a genetic mouse model of retinoblastoma, targeting HELLS decreased cellular proliferation, tumorigenesis, and morbidity and increased survival [47,50].

Glioblastoma is a fast-growing and aggressive brain tumor that contains a population of stem cell–like, highly proliferating glioblastoma stem cells (GSC). HELLS is preferentially expressed in GSC, and shRNA-mediated targeting of HELLS reduces cell cycle progression, induces replication stress, and increases DNA damage and apoptosis in GSC. In glioblastoma, HELLS interacts with the oncogenic transcription factors E2F3 and MYC to support GSC-specific proliferation and maintenance. In HELLS-deficient GSC, E2F3 and MYC bind less to target sequences, resulting in lower expression of the target genes. Targeted inhibition of HELLS by shRNA in an orthotopic xenograft model resulted in improved survival and decreased glioblastoma tumor growth. Thus, targeting HELLS in glioblastoma might offer a viable approach to disrupting the relatively undruggable MYC and E2E3 oncogenic transcription factors [37].

HELLS plays oncogenic roles in the development and progression of pancreatic cancer and serves as a biomarker of poor prognosis for pancreatic cancer. Remarkably, downregulation of HELLS reverses the malignant phenotype in pancreatic cancer [51]. HELLS is upregulated in clinical pancreatic cancer tissues, and its upregulation also correlates with advanced clinical stage and poor prognosis. HELLS-downregulated cells show increased sensitivity to cisplatin treatment in vitro and in vivo by elevating DNA damage and apoptosis. Mechanistically, HELLS overexpression inhibited transcriptional expression of the tumor suppressor TGFBR3 in this model, and downregulation of HELLS in pancreatic cancer cells led to re-expression of TGFBR3 [35].

HELLS has also been implicated in nasopharyngeal carcinoma (NPC). Elevated expression of HELLS promotes NPC progression, in part by downregulating the expression of fumarate hydratase, a component of the tricarboxylic acid cycle, through repression of its promoter [52]. It also increases the expression level of a mesenchymal marker (vimentin), suggesting contribution of HELLS to the epithelial-to-mesenchymal transition, which confers metastatic potential to cancer cells [52].

HELLS has been linked with tumorigenesis and tumor progression in lung cancer. HELLS is upregulated in lung cancer tissues, and the increased levels are associated with poor overall survival in lung cancer patients [53]. The siRNA-mediated inhibition of HELLS in HT29 and HCT116 lung cancer cell lines decreases cell proliferation and colony formation and increases G2/M cell cycle arrest [45]. HELLS is overexpressed in the breast cancer cell lines MDA-MB-231 and MCF7, where it is also involved in DNA methylation and Pol II stalling [54]. The knockdown of HELLS reduces cell growth in MDA-MB-231 cells.

**Figure 1:**
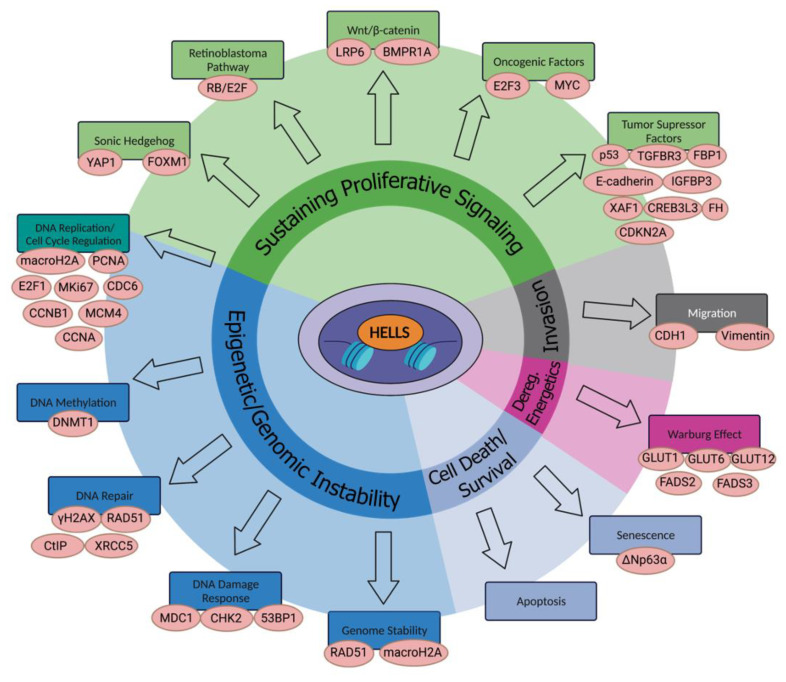
Association of HELLS with multiple pathways involved in cancer development, progression and influencing responses to therapy.

HELLS has been linked with several signaling pathways important for cancer development and progression, beyond the RB/E2F pathway (Figure 1) [49,55]. HELLS works as a coactivator of cell proliferation by binding to promoter regions of *CDC6*, *MKi67*, *PCNA*, *CCNB1*, *MCM4*, *E2F1*, and *CCNA* [37]. It acts downstream of E2F1 in osteosarcoma, glioma, and retinoblastoma [56]. In gliomas, the levels of HELLS are regulated by the LRP6-GSK3b-E2F1 axis. HELLS also interacts with E2F3A and E2F3B and cooperates with their oncogenic functions [55]. Wnt/β-catenin signaling, which maintains many types of embryonic and adult stem cells and contributes to the generation of tumor-propagating cells, regulates HELLS expression [57]. One-third of medulloblastomas evolve from an aberrant activation of the sonic hedgehog (SHH) signaling pathway. HELLS is overexpressed in SHH human and murine medulloblastoma tumors, and its expression its activated by the SHH pathway, specifically by YAP1, which acts downstream of Smoothened [58]. In head and neck squamous cell carcinoma, the oncogene FOXM1, a downstream target of the oncogenic SHH signaling pathway, upregulates HELLS, and HELLS is then responsible for promoter hypermethylation of tumor suppressors such as CDKN2A [59]. HELLS is significantly overexpressed in clinical samples of colorectal cancer. Downregulation of HELLS in colorectal cancer cells led to decreased cell proliferation, colony formation, and G2/M cell cycle arrest [45]. In keratinocytes LSH has been reported to work as a mediator of senescence bypass through the activation of the oncogene ΔNp63α. HELLS is overexpressed in HNSCC cells that shows overexpression of ΔNp63α and the knockdown of HELLS resulted in decrease of cell proliferation [60].

## 8. Conclusions and Future Perspectives

The inability to effectively cope with genotoxic stress can lead to genome instability, mutagenesis, or cell death, molecular events that play critical roles in cancer development, progression, and treatment. Chromatin creates a functional platform for tight, multi-level regulation of DNA repair and genome maintenance pathways. HELLS has emerged as an important, chromatin-based regulator of DNA repair, genome stability, and multiple cancer pathways. However, several questions remain to be addressed concerning the cellular specificity, chromatin context, and mechanistic details of HELLS-mediated genome maintenance. Is HELLS involved in DNA repair in specific cell types, such as highly proliferating cells? Does HELLS coordinate multiple DNA repair pathways? Is HELLS necessary for modulating DNA repair across the entire genome, or are there specific genes and domains that rely on HELLS function? What is the influence of HELLS-mediated DNA methylation loss on DNA repair? How does overexpression of HELLS affect DNA repair, genome stability, and responses to chemotherapy-induced genotoxic stress? Understanding the molecular functions of HELLS as they pertain to DNA repair is important for advancing our understanding of chromatin-based genome maintenance and genome instability in cancer. Future studies investigating the role of HELLS in genome maintenance pathways will advance current understanding of cancer development and progression, and will likely uncover new therapeutic approaches, such as targeting HELLS to sensitize cancer cells to chemotherapy treatments.

HELLS is emerging as an attractive potential therapeutic target because it is associated with regulation of a multitude of cancer pathways through enzymatic ATPase-mediated chromatin remodeling. Therefore, targeting HELLS could lead to simultaneous inactivation of multiple oncogenic pathways that sustain cancer proliferative potential, underlie genomic instability and collectively contribute to cancer progression and invasion. Specific small molecule inhibitors or proteolysis-targeting chimera degrader (PROTAC) could be developed to target HELLS. Several ATPase inhibitors, and degraders targeting number of SNF2 remodelers have been previously developed highlighting the feasibility of the approach [61,62,63]. The crystal structure of HELLS remains to be solved to guide the design of small molecule inhibitors targeting ATPase enzymatic activity. Using small molecules or degraders targeting HELLS in combination with chemotherapy might achieve a favorable outcome in a subset of cancers, especially those where HELLS is overexpressed and involved in cancer cell proliferation and metastasis. Future in vitro and in vivo studies will be necessary to determine the effectiveness and utility of targeting HELLS in cancer therapy and evaluating any associated toxicity and adverse effects.

## Figures and Tables

**Table 1:** Contribution of HELLS to various aspects of DNA repair and genome stability.

Model System	Defect in DNA Repair and/or DDR	DNA Damage Sensitivity	Genome Instability	Reference
LSH-deficient MEFs and MRC5 fibroblasts	Deficient phosphorylation of ɣH2AX in response to gamma irradiation Deficient repair of DSB	Increased sensitivity to gamma irradiation, hydrogen peroxide, HU, MMS	Not Determined	Burrage et al., 2012 [32]
MHCC-97L transfected with sgRNA targeting HELLS	Possible block of DNA DSB repair (assessed by ɣH2AX)	Not determined	Not determined	Law et al., 2019 [34]
HELLS siRNA in PANC-1 and BxPC-3	Increased DNA damage measured by γH2AX levels	Increased sensitivity to cisplatin 20 µM after 24 h	Not determined	Hou et al., 2021 [35]
HEK293 cells carrying ICF HELLS mutations	Delay in XRCC5 accumulation at DNA damage sites, defects in NHEJ	No detected sensitivity to olaparib, MMC, TMZ	Accumulation of γH2AX signals colocalizing with centromeric regions, chromosome abnormalities, aneuploidy	Unoki et al., 2019 [29]
HELLS siRNA in 1BR-hTert, HeLa	Increase of γH2AX, decrease of Rad51 foci (also in U2OS cells) Deficient HR in heterochromatin of G2/M cells.	Mild sensitivity to olaparib	Increase in spontaneous micronuclei, decrease in sister chromatid exchange in HeLa cells 12 h after 3 Gy	Kollárovič et al., 2019 [31]
LSH siRNA in LPS-stimulated mouse lymphocytes	Increase of ɣH2AX, degradation of nascent DNA at stalled replication forks through macroH2A deficiency	Increased sensitivity to HU and APH	Increase in sister chromatid exchange, fragile telomeres, and chromosome aberrations	Xu et al., 2021 [36]
HELLS siRNA in GSC387 and GSC3565	Increased endogenous DNA damage as measured by ɣH2AX	Not determined	Not determined	Zhang et al., 2019 [37]
LSH siRNA in U2OS cells	Reduced NHEJ	Not determined	Not determined	He et al., 2020 [38]
MUS-30 knockout in *Neurospora Crassa*	Possibly defect in S-phase associated DNA repair	Hypersensitivity to MMS and TBP	Not determined	Basenko et al., 2016 [19]
IRC-5 knockout in *Saccharomyces cerevisiae*	Defect in cohesion loading	Sensitivity to MMS	Instability of rDNA loci	Litwin et al., 2017 [20]
DDM1 knockout in *Arabidopsis Thaliana*	Deficient BER and NER repair capacity	Sensitivity to MMS	Not determined	Yao et al., 2012 [22]

Abbreviations: BER, base excision repair; DDR, DNA damage response; DSB, double-strand breaks; LPS, lipopolysaccharide; MEF, mouse embryonic fibroblast; MMS, methyl methanesulfonate; NER, nucleotide excision repair; NHEJ, nonhomologous end joining; HU, hydroxyurea; MMC, mitomycin C; TMZ, temozolomide; APH, aphidicolin; TBP, tert-Butyl hydroperoxide.

## Data Availability

Not applicable.
